# Influence of Oxygen Vacancy Density on the Polaronic Configuration in Rutile

**DOI:** 10.3390/ma11112156

**Published:** 2018-11-01

**Authors:** Rulin Liu, Liang Fang, Yue Hao, Yaqing Chi

**Affiliations:** 1 Institute for Quantum Information & State Key Laboratory of High Performance Computing, College of Computer, National University of Defense Technology, Changsha 410073, China; 2 State Key Discipline Laboratory of Wide Bandgap Semiconductor Technologies, School of Microelectronics, Xidian University, Xi’an 710071, China; yhao@xidian.edu.cn; 3 College of Computer, National University of Defense Technology, Changsha 410073, China; yqchi@nudt.edu.cn

**Keywords:** rutile, oxygen vacancies, polarons, electronic structure, DFT + *U*

## Abstract

Polaronic configurations that were introduced by oxygen vacancy in rutile TiO_2_ crystal have been studied by the DFT + *U* method. It is found that the building block of TiO_6_ will expand when extra electron is trapped in the central Ti atom as polaron. With manually adjusting the initial geometry of oxygen vacancy structure, a variety of polaronic configurations are obtained after variable-cell relaxation. By calculating different sizes of supercell model, it is found that the most stable configuration can be influenced by the density of oxygen vacancy. With increasing interaction between vacancies, the most stable polaronic configuration change from small polaronic configuration to mixed configuration.

## 1. Introduction

TiO_2_ is a material that is widely used in various applications such as photocatalysis [1], solar cell [2], and resistive random access memory device (RRAM) [3,4]. These technological uses rely mainly on the control of defects and charge carrier. Take RRAM as an example, the resistance of device changes under external electric filed, which cased by defect diffusion. Among all the intrinsic defects, oxygen vacancy (V_O_) is the most important type which has been studied by a variety of experimental techniques. As defects in crystal exhibit special characteristics of electron spins, ionization, and excitation. Electron spin resonance (ESR) experimental results reveal the existence of Ti^3+^, which is introduced by oxygen vacancies [5,6]. The corresponding localized states at about 1 eV below conduction band minimum (CBM) in the band gap have been detected by infra-red (IR) spectrum [7].

Numerious theoretical calculations have also been performed to study detailed properties of oxygen vacancy in rutile [8,9,10,11,12,13,14,15,16]. Standard density functional theory (DFT) method gives a completely delocalized configuration without any localized states in the band gap with Fermi level lies in the conduction band [17]. The corresponding bandgap is also highly underestimated. The failure of standard DFT method is caused by its improperly handling of electron correlation, also called self-interaction error (SIE) [18], which is caused by the underestimation of electron localization during calculation. To tackle this problem, many post-DFT methods, including DFT + *U* [15,19,20,21,22,23], Hybrid DFT functionals [9,12,13,14,24,25,26], Green’s function, and screened Coulomb interaction (GW) correction [27,28] have been employed to recover the band structure behavior. For all these correction methods, electron correlations are included in mean-filed Hubbard *U* in DFT + *U* [29], or a portion of exact exchange in Hartree-Fock (HF) method [30], or calculated by one-electron Green’s function in GW method [31].

The electron distribution is highly related to the calculation methods, especially the value of on-site Coulomb parameter for DFT + *U* calculation and the ratio of HF exchange functional in hybrid functionals [17]. Previous calculations have controversy on the electron distribution of oxygen vacancy in rutile. Different configurations, including localized polaronic states [9,15], hybrid states [8], delocalized states [15], and singlet states [9,16] have been obtained by different calculation methods. Among all of these results, small polaronic configuration is energetically favored than singlet states and delocalized state.

In this article, calculations have been done to study different polaronic configurations within different size of rutile supercell models. The SIE problem is corrected by employing Hubbard correction with DFT + *U* method and the Coulomb repulsion potential term were calculated by the linear response method. It is found that the most stable configuration can be affected by the density of oxygen vacancy, which changes from small polaronic configuration in 3 × 3 × 5 supercell to mixed configuration in 2 × 2 × 3 supercell due to increasing interaction between vacancies.

## 2. Computational Methods

Calculations were performed within the framework of density functional theory (DFT) [32] using QUANTUM ESPRESSO (Version 6.1) [33]. Oxygen vacancy structure were constructed from a 3 × 3 × 5 supercell (270 atoms), 3 × 3 × 4 supercell (216 atoms) and a 2 × 2 × 3 supercell (72 atoms) by removing one oxygen in crystal grid. The Perdew-Burke-Ernzerhof (PBEsol) [34,35] GGA exchange correlation functional was applied and the PBEsol pseudopotentials were supplied by SSSP [36,37]. Energy cutoff of 650 eV were set for variable-cell relaxation process with Monkhorst-Pack k-point sampling in the Brillouin zone were chosen as Gamma for 216-atom and 270-atom supercell and 2 × 2 × 2 for the 72-atom supercell. The cutoff energy (*E_cut_*) and *k*-sampling have been carefully examined to ensure total energy (*E_tot_*) convergence with criterion of d*E_tot_*/d(ln*E_cut_*) < 0.01 eV per atom. All calculations, if not specified, were set without geometrical symmetry or spin constraints.

In this paper, Hubbard *U* correction had been employed to tackle the self-interaction error. The value of *U* was calculated by the linear response method [38,39], which needs a variety calcualtions with different perturbation on single atom and the *U* can be obtained by linear fitting of electron occupations. As a charge transfer insulator, the conduction band minimum (CBM) of rutile is dominated by Ti-3*d* orbitals and the valence band maximum (VBM) is dominated by O-2*p* orbitals. Previous DFT + *U* studies point to the fact that correction on Ti-3*d* orbitals underestimates the bandgap even at a high value [23,40], which is enough for Mott insulators with CBM and VBM composed by same angular momentum orbitals. Reference [41] points out that there exists residual self-interaction within O-2*p* orbital and a number of literatures have employed $U_{O}^{2p}$ correction for better description of electronic structure [20,40,41,42]. In this paper, Hubbard correction $U_{O}^{2p}$ together with $U_{Ti}^{3d}$ are used in calculation. As O-2*p* orbital is nearly full in rutile crystal, $U_{O}^{2p}$ calculated by linear response method is larger than its actual value [43]. So, we choose $U_{O}^{2p}$ by fitting band gap with experimental value. The *U* value can be obatined by the following process:

1. Calculate linear response on single Ti atom in perfect rutile crystal without setting any *U* value. A value of $U_{Ti}^{3d}$ = 4.2 eV can be obtained for Ti-3*d* orbitals.
2. Calculate band gap with $U_{Ti}^{3d}$ = 4.2 eV and variable $U_{O}^{2p}$. A value of $U_{O}^{2p}$ = 4.5 eV fits the experimental value.
3. Re-calculate linear response of single Ti atom in perfect rutile crystal with $U_{O}^{2p}$ = 4.5 eV. A new value of about $U_{Ti}^{3d}$ = 4.0 eV can be obtained with this $U_{O}^{2p}$. The use of $U_{O}^{2p}$ will lower electron population on Ti atoms, which decrease the linear response result from $U_{Ti}^{3d}$ = 4.2 eV to 4.0 eV.

The band gap with $U_{O}^{2p}$ = 4.5 eV and $U_{TI}^{3d}$ = 4.0 eV correction is 2.979 eV, which is consistent with the experimental value of 3.0 eV [44,45]. The optimized lattice constants gave *a* = 4.562 Å and *c* = 3.001 Å, within 1.5% of the experimental data [46]. In addition, as the value of *U* is related to the chemical environment, each atom should be separately calculated in certain defect structure, which will be discussed in the following text.

**Figure 1:**
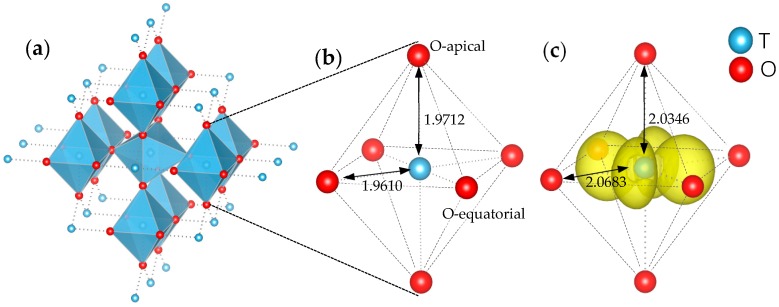
(**a**) Rutile crystal in view of TiO_6_ building blocks. (**b**,**c**) Change of Ti–O bond length before and after a polaron is trapped at the central Ti atom. Yellow lobes correspond to net spin with density iso-surface of 0.01 e·A^−3^.

TiO_6_ octahedra is basic building block in rutile with each O atom is 3-coordinated and each Ti is 6-coordinated. Polaron trapping at single Ti crystal grid of perfect rutile supercell was calculated by setting an extra electron in relaxation. As shown in Figure 1, all the six chemical bonds of Ti–O are elongated when polaron is formed on central Ti, with equatorial bond increased from 1.9610 Å to 2.0683 Å and apical bond increased from 1.9712 Å to 2.0346 Å. This expansion weakens the Jahn-Teller distortion. Inspired by this phenomenon, we can construct special polaronic configurations with electron trapped at arbitrary Ti sites by outwardly moving the coordinated O atoms with 20% of bond length in starting structure of variable-cell relaxation.

## 3. Results and Discussion

**Figure 2:**
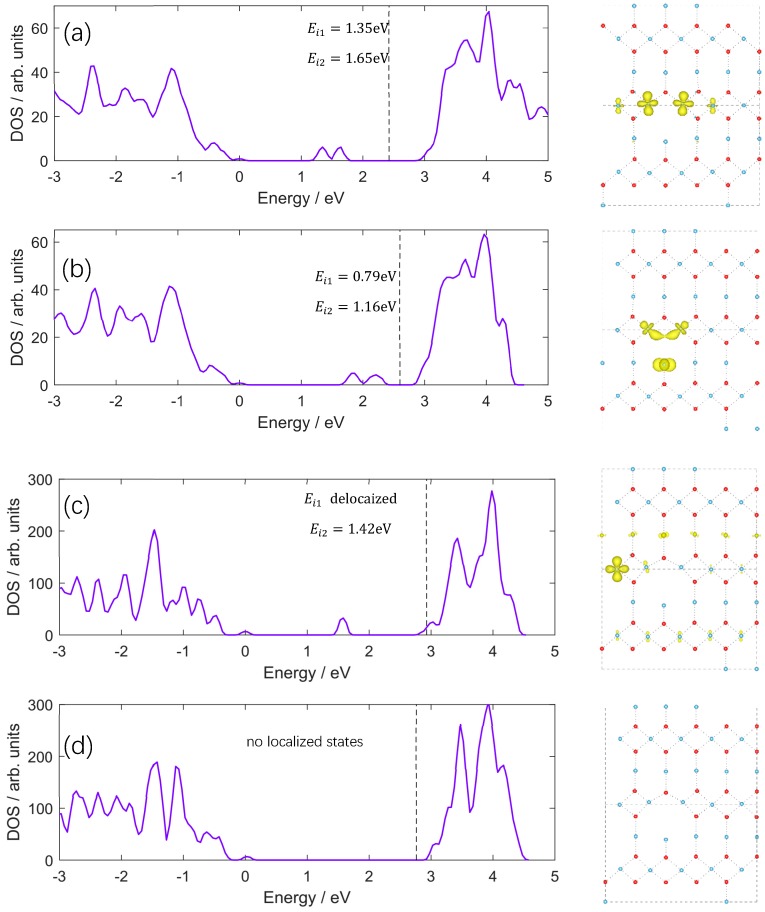
Density of state (DOS) of polaronic configurations of 3 × 3 × 5 supercell and corresponding atomic structures of VO-(110) plane. The vertical dashed line denotes the Fermi level, *E_i_*_1_ and *E_i_*_2_ represent the relative energy of polaronic states and conduction band minimum. Subgraph (**a**) is small polaronic configuration of two electrons are each localized at single Ti site forming two small polarons. (**b**) is mixed configuration of one of the two electrons are shared by two equatorial Ti atoms forming a hybrid state (also called large polaronic state [48]) and the other is localized at apical Ti as small polaron. This configuration is called mixed configuration in the following text. (**c**) is partly delocalized configuration of one electron is trapped at Ti as a small polaron with only one localized state in the bandgap. The other electron is delocalized and the Fermi level moves up into the bottom of conduction band. (**d**) Singlet state with no net spin and localized states in the bandgap. Yellow lobes on Ti atoms correspond to net spin with density iso-surface of 0.01 e·A^−3^. All the solutions are sorted by total energy and labeled in alphabetical order.

One oxygen vacancy in rutile will release two nominal electrons to crystal. These electrons can be distributed at various crystal site with or without spin polarization. Figure 2 shows four typical polaronic configurations of 3 × 3 × 5 supercell, which is in view of (110) plane. Subgraph (a) to (d) are small polaronic configuration, mixed configuration, delocalized configuration, and singlet configuration, respectively. The density of state (DOS) in (a) has two deep feature peaks that are located in the band gap at 1.65 and 1.35 eV below conduction band minimum, which are caused by polarons. In (b), these two peaks locate at a rather shallower position of 1.16 and 0.79 eV. One of these peaks in (c) disappears with Fermi level shifts up into the conduction band indicates the electron can be free carrier in this configuration. The singlet configuration (d) shows no localized states. Among all the configurations in Figure 2, (a) is the most stable configuration in energy than (b–d), which is consistent with former literatures [8,9,15].

Under the ionic limit, Ti atoms in rutile donate four electrons to O atoms, resulting in the stoichiometrically nominal ion of Ti^4+^ and O^2−^. In the concept of theoretical calculation, such as Bader population analysis, the electron population deviates from ionic limit. Our Bader calculations show that each Ti ion give up 2.5 electrons in rutile and 2.15 electrons in Ti_2_O_3_, which is consistent with former literature [47]. It can be seen that when a Ti atom has a Bader charge of +2.5e, it corresponds to the nominal Ti^4+^ and Bader charge of +2.15e to nominal Ti^3+^ cation.

When an oxygen atom is removed from lattice grid, two electrons is released to the crystal and the three adjacent Ti atoms move outwardly due to electrostatic interaction. In Table 1, Bader population analysis of Index-(a) reveals that the equatorial Ti adjacent to oxygen vacancy owns about 0.35 electron more than Ti atoms in normal lattice grid, which indicates that the two Ti^4+^ tend to be reduced to Ti^3+^. For Index-(b) of mixed configuration, the apical Ti is reduced to Ti^3+^, while the other two equatorial Ti atom share one electron forming a hybrid state.

**Figure 3:**
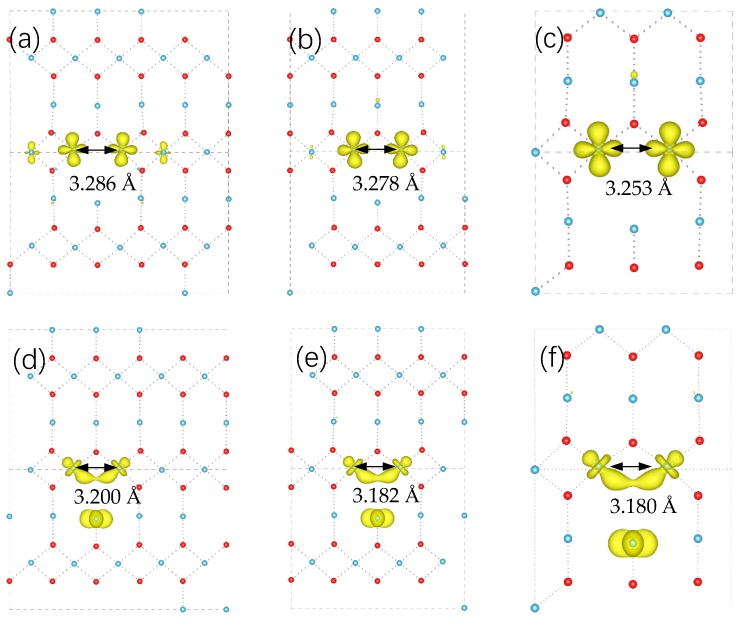
Polaronic structure of oxygen vacancy with different size of supercell. (**a**,**d**) for 3 × 3 × 5 supercell, (**b**,**e**) for supercell 3 × 3 × 4, (**c**,**f**) for 2 × 2 × 3 supercell. In all of the configurations, the distances between two equatorial Ti atoms get shorter with gradually decreasing the size of supercell along *c* axis.

Polaronic configurations of three different supercell size with single oxygen vacancy are shown in Figure 3. It can be found that as the size of supercell change from 3 × 3 × 5 to 2 × 2 × 3, the distance between polarons get smaller.

As the value of *U* is related to the chemical environment, each atom should be separately calculated in defect structure. Table 2 lists all the renewed *U,* which is calculated by linear response method. It can be seen that the existence of oxygen vacancy dramatically affects the *U* values of adjacent Ti atoms. While for normal six-coordinated Ti atoms, the *U* values are barely affected, which is always 4.0 eV. In addition, the *U* value also changes with different polaronic configurations, especially for equatorial Ti in mixed configuration. For 2 × 2 × 3 supercell, the *U* value of 3.87 eV is even smaller than comparing with normal Ti, which indicates a stronger delocalization character. As a result, this small *U* lead to a hybrid state of one electron shared by two equatorial Ti atoms.

By re-calculating the total energy with new *U*, we find the relative stability of these polaronic configurations change with supercell size. For 3 × 3 × 5 supercell, mixed configuration in Figure 3b is about 1.616 eV higher in energy than small polaronic configuration in Figure 3a. For 3 × 3 × 4, this difference reduces to 0.105 eV. For 2 × 2 × 3 supercell, the opposite result shows the mixed configuration in Figure 3f is more stable with 0.517 eV than Figure 3c. As the value *U* can reflect the degree of electron repulsion, the change of the most stable configuration can be interpreted as the interaction between vacancy defects.

**Figure 4:**
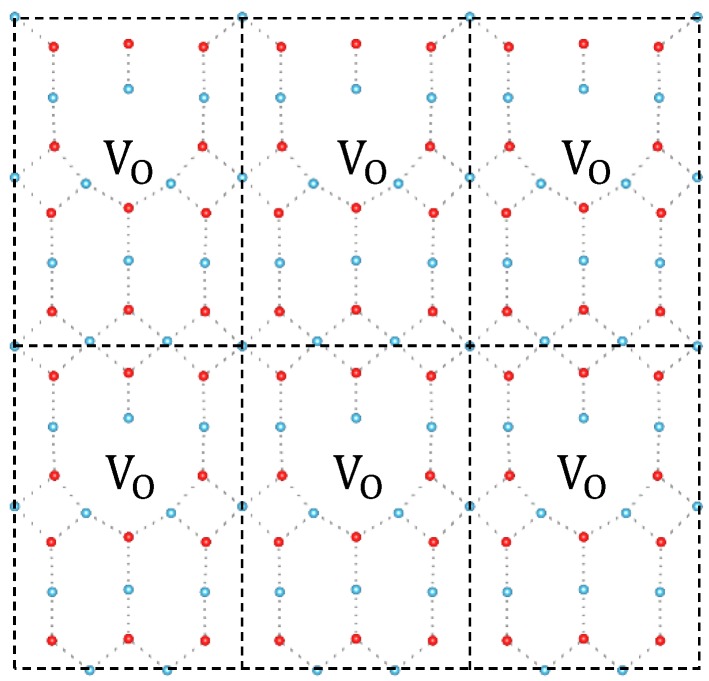
Schematic structure of 2 × 2 × 3 oxygen vacancy structure in periodical method. In the framework of the density functional theory (DFT) method, all calculations are performed in infinite size, which is constructed through periodic replication of input structure.

As shown in the TiO_6_ building block of Figure 1, three adjacent Ti near O can be divided into two groups that two-equal equatorial Ti form shorter Ti–O bonds and one apical Ti form longer bond. With shorter distance from the vacancy, two equatorial Ti attract more electrons than the apical Ti in a large supercell (diluted oxygen vacancy density). As periodical supercell method is used in DFT calculation, Figure 4, the influence of mirrored defects in periodical supercells cannot be ignored in small model, which can be seen as density effect of oxygen vacancy. In Figure 3c, the distance of two equatorial Ti atoms is shorter than Figure 3a that is caused by the Coulomb repulsion between mirrored polarons, which leads an electron to transfer to apical Ti atom. In this situation, polaron trapping on the apical Ti is energetically favored. As the transition of stable configuration occurs during decreasing the supercell size, the corresponding ionization energy of localized polaron also changes from 1.35 eV to 0.79 eV (from Figure 2a to Figure 2b), which is consistent with Infra-red (IR) absorption spectrum with variable oxygen vacancy [7].

## 4. Conclusions

Based on the DFT + *U* method, the polaronic configurations that induced by oxygen vacancy in rutile crystal are studied. Electrons introduced by oxygen vacancy in rutile can be distributed at various crystal site with or without spin polarization, that polaron can be formed at an arbitrary Ti site by elongating coordinated chemical bonds in starting structure of geometrical relaxation. It is found that the value of Coulomb respulsion parameter *U* is sensitve with polaronic configurations and oxygen vacancy density. The most stable configuration changes from small polaronic configuration in 3 × 3 × 5 supercell to mixed configuration in 2 × 2 × 3 supercell due to increasing interaction between vacancies with higher oxygen vacancy density. The evolution of stable configuration with vacancy density is consistent with IR absorption spectrum. In addition, our results reveal that the size of supercell used in theoretical calculation for light doping should be carefully tested to avoid the finite size effect from mirrored defects.

## Figures and Tables

**Table 1:** Bader charge of small polaronic configuration and mix configuration of 3 × 3 × 5 supercell in Figure 2. The numbers in parentheses give the difference of electron population of Ti around oxygen vacancy and normal grid Ti.

Index	Normal Ti	Equatorial Ti	Apical Ti
(a)	2.50	2.15 (0.35)	2.42 (0.08)
(b)	2.50	2.29 (0.21)	2.12 (0.38)

**Table 2:** Hubbard *U* parameter of polaronic configurations in Figure 3. All of the *U* values are calculated by linear response method. Index (a–c) for small polaronic configurations and (d–f) for mixed polaronic configurations of 3 × 3 × 5, 3 × 3 × 4 and 2 × 2 × 3 supercell.

Index	*U* of Equatorial Ti	*U* of Apical Ti	Index	*U* of Equatorial Ti	*U* of Apical Ti
(a)	4.81 eV	4.00 eV	(d)	4.66 eV	5.18 eV
(b)	4.80 eV	4.01 eV	(e)	4.01 eV	5.18 eV
(c)	5.03 eV	4.12 eV	(f)	3.87 eV	5.32 eV
